# Forest Biomass as a Promising Source of Bioactive Essential Oil and Phenolic Compounds for Alzheimer’s Disease Therapy

**DOI:** 10.3390/ijms23158812

**Published:** 2022-08-08

**Authors:** Patrícia Moreira, Patrícia Matos, Artur Figueirinha, Lígia Salgueiro, Maria Teresa Batista, Pedro Costa Branco, Maria Teresa Cruz, Cláudia Fragão Pereira

**Affiliations:** 1 CNC—Center for Neuroscience and Cell Biology, CIBB—Center for Innovative Biomedicine and Biotechnology, University of Coimbra, 3004-504 Coimbra, Portugal; 2 Faculty of Pharmacy, University of Coimbra, 3000-548 Coimbra, Portugal; 3 LAQV, REQUIMTE, Faculty of Pharmacy, University of Coimbra, 3000-548 Coimbra, Portugal; 4 CIEPQPF, Research Center for Chemical Processes Engineering and Forest Products, Faculty of Pharmacy, University of Coimbra, 3000-548 Coimbra, Portugal; 5 RAIZ—Forest and Paper Research Institute, 3800-783 Aveiro, Portugal; 6 Faculty of Medicine, University of Coimbra, 3000-548 Coimbra, Portugal

**Keywords:** essential oil, phenolic extracts, eucalyptus, Alzheimer’s disease

## Abstract

Alzheimer’s disease (AD) is the most common neurodegenerative disorder affecting elderly people worldwide. Currently, there are no effective treatments for AD able to prevent disease progression, highlighting the urgency of finding new therapeutic strategies to stop or delay this pathology. Several plants exhibit potential as source of safe and multi-target new therapeutic molecules for AD treatment. Meanwhile, *Eucalyptus globulus* extracts revealed important pharmacological activities, namely antioxidant and anti-inflammatory properties, which can contribute to the reported neuroprotective effects. This review summarizes the chemical composition of essential oil (EO) and phenolic extracts obtained from *Eucalyptus globulus* leaves, disclosing major compounds and their effects on AD-relevant pathological features, including deposition of amyloid-β (Aβ) in senile plaques and hyperphosphorylated tau in neurofibrillary tangles (NFTs), abnormalities in GABAergic, cholinergic and glutamatergic neurotransmission, inflammation, and oxidative stress. In general, 1,8-cineole is the major compound identified in EO, and ellagic acid, quercetin, and rutin were described as main compounds in phenolic extracts from *Eucalyptus globulus* leaves. EO and phenolic extracts, and especially their major compounds, were found to prevent several pathological cellular processes and to improve cognitive function in AD animal models. Therefore, *Eucalyptus globulus* leaves are a relevant source of biological active and safe molecules that could be used as raw material for nutraceuticals and plant-based medicinal products useful for AD prevention and treatment.

## 1. Introduction

Alzheimer’s disease (AD) is a multifactorial age-related neurodegenerative disorder that is characterized by loss of memory and impairment of other cognitive functions as well as behavioral alterations [1,2]. AD is the most common form of dementia in the elderly affecting 50 million people worldwide and is expected to impact on 152 million people in 2050 [3], highlighting the urgency of developing disease-modifying strategies able to prevent or delay its progression. The characteristic neuropathological hallmarks, which have been implicated in AD pathophysiology, are the intracellular accumulation of neurofibrillary tangles (NFTs) composed of hyperphosphorylated tau protein and the extracellular deposition of amyloid-β (Aβ) peptide in senile plaques [4]. In addition to formation and deposition of Aβ and hyperphosphorylated tau, several other molecular alterations have been described in AD, including neuronal dystrophy, synaptic loss, oxidative stress, inflammation, and depletion of acetylcholine (ACh) levels, among many others [5]. In the last decades, several efforts have been made towards the identification of novel therapeutic targets in AD because the treatments approved by Food and Drug Administration (FDA) and European Medicines Agency (EMA) only alleviate symptoms without altering the progression of the disease and also have multiple side effects. Interestingly, two of the five approved drugs are from natural origin (galantamine and physostigmine-derivative rivastigmine) [6]; therefore, the relevance of plants as sources of new safe and multi-target therapeutic agents for AD has stimulated the research worldwide [7,8,9]. In accordance, over the past 20 years, about 50% of the over 1000 different compounds that have been studied as candidates for AD treatment are molecules obtained from natural sources, and some have already been tested in clinical trials [10,11]. *Eucalyptus globulus* (*E. globulus*) is a tree native to Australia and extensively cultivated in many countries of Europe, including Portugal [12]. The exploration of eucalyptus by the pulp, paper, and wood industry generates large quantities of residual biomass (bark, leaves, and branches) whose valorization can represent a significant contribution to the circular economy. The potential of some by-products of *E. globulus* as a source of bioactive compounds has been demonstrated. In fact, *E. globulus* leaves are traditionally used for treatment of respiratory threats. Furthermore, several compounds obtained from *E. globulus* leaves, such as essential oils (EOs) and phenolic compounds, have been shown to be important antimicrobial agents as well as to exhibit antioxidant and anti-inflammatory properties, among other relevant biological activities [12,13], reinforcing the interest in deepening further studies focused on this by-product. In fact, due to the already demonstrated properties, these compounds have been used against bacteria and fungi infections, for pain relief, and to deal with immune system-related diseases besides respiratory problems [14]. Additionally, there are some evidence that these compounds could be applied to the skin to deal with conditions, which is supported by our recent work demonstrating their beneficial effect against several skin alterations such as aging and pigmentation [15]. The therapeutic potential for AD of some of these compounds was also reported in a few studies due to their inhibitory effect on acetylcholinesterase (AChE) and neuroprotective effects [13,16]. This review summarizes the chemical composition and the potential of EOs and phenolic compounds extracted from *E. globulus* leaves, particularly the major components, as promising therapeutic agents for AD.

## 2. Chemical Composition of Essential Oil and Phenolic Compounds from *E. globulus* Leaves

The International Standard Organization on Essential Oils (ISO 9235: 2013) and the European Pharmacopoeia define an EO as the product obtained from plant raw material by hydrodistillation, steam distillation or dry distillation, or by a suitable mechanical process (for *Citrus* fruits). EOs are usually complex mixtures of volatile compounds present in different concentrations [17]. Monoterpenes and sesquiterpenes are usually the main groups found in EOs, and in some cases, phenylpropanoids are also important components [18]. The yield and chemical composition of EOs depend on several extrinsic (ecological and environmental aspects) and intrinsic (sexual, seasonal, ontogenetic, and genetic variations) factors [19]. Generally, the chemical characterization of EOs is performed by gas chromatography-mass spectrometry (GC-MS) techniques, and the quality of EOs is evaluated by comparison with analytical monographs published by the European Pharmacopoeia. This compendium determines the chromatographic profile of the EO obtained from *E. globulus* leaves by gas chromatography, establishing the range of the main constituents that the EO should contain at least 70% 1,8-cineole, 4–12% limonene, 1–9% α-pinene, less than 1.5% β-pinene and α-phellandrene, and less than 0.1% camphor. Concerning the EO yield, it is reported to range from 1.5 to 3.5%. Several chemical profiles have been described in the literature, with significant variations of the main compounds, as well as the EO yield, which can change according to several factors, namely the geographical region of the plants, the maturity state, and the condition of the leaves (fresh or dry), as summarized in the Table 1. According to these studies, the EOs of *E. globulus* leaves from several world regions are enriched in 1,8-cineole, and high content in this compound was reported in EOs obtained from this specie planted in Argentina (98.9%) [20], Tunisia (95.6%) [21], Italy (95.5%) [22], Brazil (90.0%) [23], Australia (90.0%) [24], India (85.0%) [25], Ethiopia (81.6%) [26], Morocco (80.0%) [27], Algeria (78.5%) [28], and Portugal (74.6%) [29]. Only two EOs have a completely different chemical profile with absence or very low levels of 1,8-cineole [30,31]. 

According to the Encyclopedia of Food Sciences and Nutrition, phenolic compounds present hydroxylated aromatic rings, in which the hydroxyl group is directly attached to the phenyl, substituted phenyl, or other aryl group [117]. Phenolic compounds are a large group of secondary metabolites produced by plants in response to environmental stresses, such as pathogen infection, high light, low temperatures, nutrient deficiency, and predators. Plants constitutively contain these compounds, which are a varied group of phytochemicals [118]. Phenolic compounds can be divided in several classes, namely phenolic acids (hydroxycinnamic acids, hydroxybenzoic acids), flavonoids, and tannins [119]. Usually, the analysis of the phenolic compounds is performed by high-performance liquid chromatography (HPLC), which offers high sensitivity and great efficiency, but gas chromatography and capillary electrophoresis can also be used. Different detection systems can be combined with these techniques, and mass spectrometry is the preferred system [120]. Regarding the phenolic compounds from *E. globulus* leaves, the chemical composition is very heterogeneous and can vary according to geographical region and the extractive solvent, as reported in several studies (Table 2). The predominant phenolic compounds from *E. globulus* leaves are phenolic acids, namely the ellagic acid and flavonoids, particularly of flavonol subclass, specifically quercetin and its glycoside rutin. Ellagitannins are predominant among the less abundant phenolic compounds in *E. globulus* leaves.

**Figure 1:**
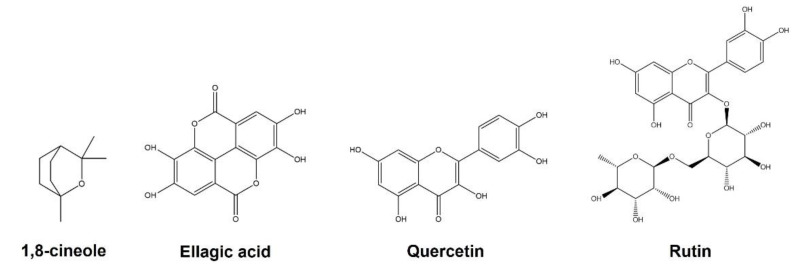
Major compounds found in essential oils and phenolic extracts obtained from *E. globulus* leaves.

The more abundant compounds found in EOs and phenolic extracts obtained from *E. globulus* leaves (Figure 1) play an important role in their biological activities. The research of *E. globulus* has mainly focused on the composition and biological activities of EOs obtained from leaves, and only few studies disclosed the chemical composition of the leaves’ phenolic extracts and their biological properties. Therefore, since *E. globulus* extracts and EOs obtained from leaves are rich in bioactive compounds, their potential for formulation of food and plant-based medicinal products should be explored. However, the analysis of the biological properties of extracts and/or isolated compounds is a key step in assessing their potential of valorization.

## 3. Role of Essential Oil and Phenolic Compounds from *E. globulus* Leaves in Alzheimer’s Disease

Table 3 lists neuroprotective effects of EO, phenolic extracts and its major constituents obtained from *E. globulus* leaves against various neurodegeneration model systems.

### 3.1. Aβ Formation and Tau Hyperphosphorylation

**Figure 2:**
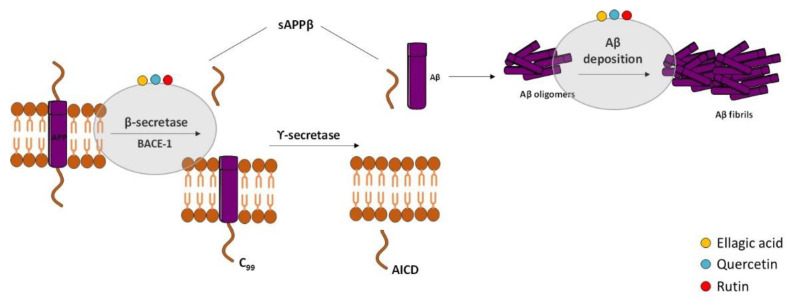
Effect of compounds obtained from *E. globulus* leaves in the amyloidogenic pathway and in the formation of amyloid-β (Aβ) in AD. The amyloidogenic pathway is initiated with the enzymatic breakdown of amyloid precursor protein (APP) by β-secretase enzyme followed by catalytic cleavage of APP by γ-secretase to originate non-soluble protein or Aβ. Aβ oligomerization and accumulation leads to synaptic dysfunction and neurodegeneration.

According to the “Amyloid Cascade Hypothesis”, accumulation and oligomerization of Aβ peptide in the brain plays a major role in AD pathophysiology [131]. Aβ is a short fragment formed by the amyloidogenic proteolytic cleavage of the amyloid precursor protein (APP) [132] (Figure 2), which exhibits toxic effects on neuronal and glia cells in both oligomeric and fibrillar forms. Therefore, several approaches have been designed to decrease Aβ peptide formation from APP, and the most studied targets are β-secretase (BACE) and the γ-secretase complex. APP cleavage is performed by these two enzymes at variable sites to form numerous fragments of Aβ [133,134]. There are two isoforms of BACE [132]: BACE-1 [135] and BACE-2 [136]. The inhibition of BACE-1 is the most attractive therapeutic approach in AD because Aβ production from APP cleavage in the brain mainly results from the action of this β-secretase isoform. The membrane fragment formed upon BACE1 action is then cleaved by γ-secretase, generating Aβ fragments, namely Aβ1-40 and Aβ1-42 [137]. The inhibition of γ-secretase is also a valuable strategy but is less attractive than β-secretase due to fact that it is a multiprotein complex. Unfortunately, serious side effects were revealed in the clinical trials performed with secretase inhibitors [138,139] since the inhibition of these two enzymes can interfere with the processing of other substrates [140,141]. For example, γ-secretase inhibition has adverse side effects on Notch signaling that may cause severe gastrointestinal toxicity and the β-secretase inhibition can affect negatively the central or peripheral myelinization. Besides secretases inhibitors and modulators aimed to reduce Aβ formation, there are other therapeutic strategies under development to halt AD progression, such as prevention of Aβ oligomerization and aggregation into plaques, Aβ vaccination to promote Aβ clearance, and inhibition of its accumulation [142]. In fact, recently, FDA approved the commercialization of a new drug for AD treatment, the aducanumab, which is an antibody capable of removing Aβ plaques from the brain and the first drug capable of interfering with the neurodegenerative process of the disease.

**Figure 3:**
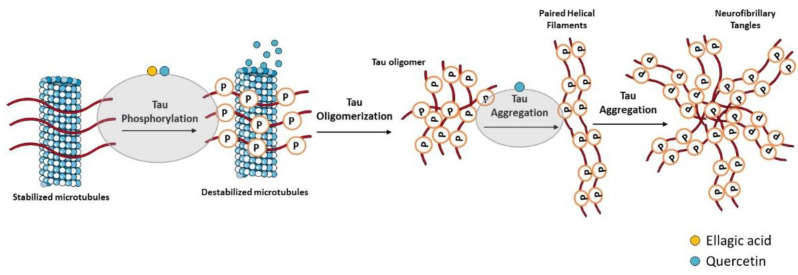
Effect of compounds obtained from *E. globulus* leaves on tau aggregation and formation of neurofibrillary tangles (NFTs) in AD. Irregular phosphorylation of tau proteins destabilizes microtubules, leading to the formation of insoluble tau oligomers, which then accumulate to generate protomers. Then, two twisted protomers originate paired helical filaments, which after aggregation lead to the formation of NFTs. These intracellular structures are involved in synaptic and neuronal dysfunction, thus contributing to cognitive decline in AD.

Hyperphosphorylated tau-enriched NFTs are another neuropathological hallmark of AD. Under physiologic conditions, tau is the principal microtubule (MT)-associated protein that cooperates with tubulin to regulate MTs stability, which is crucial to axonal transport and thus to neuronal functioning [143]. In AD, hyperphosphorylated tau loses the capacity to bind MTs and forms NFTs that contribute to the neurodegenerative process [144,145] (Figure 3). Overproduction of inflammatory mediators has been shown to activate kinases such as cyclin-dependent kinase-5 (CDK-5) and glycogen synthase kinase-3β (GSK-3β), which consequently lead to tau phosphorylation [146,147]. In AD, GSK-3β plays a crucial role in tau hyperphosphorylation [148], but it was also demonstrated to contribute to Aβ aggregation and deposition into senile plaques [149]. With this in mind, GSK-3β inhibitors could represent a promising treatment strategy for AD.

In the last years, some studies revealed that phenolic compounds can interfere with both amyloid and tau pathologies, supporting their beneficial role in AD. However, there is no information in the literature about the effect of EO from *E. globulus* leaves and its major compound 1,8-cineole on AD. Ellagic acid was found as a potential BACE-1 inhibitor as well as a protective strategy against Aβ deposition and tau hyperphosphorylation. In a screening for anti-dementia agents from natural products, Kwak and collaborators (2005) reported that ellagic acid was a moderate BACE-1 specific inhibitor [150] and in vitro studies showed that ellagic acid promoted a significant loss of oligomers levels and was able to prevent Aβ-induced toxicity [151,152]. Accordingly, ellagic acid treatment in a sporadic AD rat model induced by streptozotocin (STZ) administration markedly decreased brain Aβ levels, suggesting its potential to delay amyloidogenesis [153]. Finally, it was reported that ellagic acid decreased APP and BACE-1 expression levels as well as Aβ deposition in the hippocampus of APP/PS1 transgenic mice, a model of familial AD [154]. This study also described the inhibition of tau hyperphosphorylation by ellagic acid mediated by the activation of the protein kinase B (Akt)/GSK-3β signaling pathway.

Regarding quercetin and rutin, several studies identified quercetin and rutin as BACE-1 inhibitors and provided strong evidences that both compounds are able to reduce Aβ deposition and quercetin to decrease tau hyperphosphorylation and aggregation, proving its neuroprotective effects. In different in vitro AD models, both compounds showed to prevent Aβ fibrils formation and cytotoxicity [155,156,157,158], and rutin was identified as a BACE-1 inhibitor that specifically prevents APP cleavage, decreasing production of the sAPPβ fragment [155,159,160]. In primary cortical neurons, quercetin was also described to act as a potent BACE-1 inhibitor and to decrease Aβ levels [161]. However, an in vitro study of Paris and co-authors provided evidences that quercetin inhibits Aβ and sAPPβ production by regulating BACE-1 expression and not by acting directly as an inhibitor of its activity [162]. Furthermore, a combined in vitro cell-based/in silico screening reported that quercetin shows potent Aβ anti-aggregation activity [163]. Moreover, in HT22 hippocampal neurons as well as in differentiated SH-SY5Y, quercetin reduced okadaic acid (OA)-induced tau hyperphosphorylation, inhibited the activity of CD-K5, attenuated the rise of intracellular calcium, and inhibited neuronal apoptosis via suppression of phosphoinositide 3-kinase (PI3K)/Akt/GSK-3β, mitogen-activated protein kinases (MAPKs), Bcl-2-associated X (BAX), and caspase-3 activities as well as nuclear factor-κB (NF-κB) activation [164,165,166]. Furthermore, quercetin was found to prevent tau phosphorylation through AMP-activated protein kinase (AMPK) activation and GSK-3β inhibition in OA-treated SH-SY5Y cells and in the hippocampus of mice fed with a high-fat diet [167]. Quercetin also inhibited Aβ fibrillization but not its toxic oligomerization in a *C. elegans* model of Aβ deposition [168] by activation of macroautophagy and proteasomal degradation pathways [169]. In accordance, senile plaques were reduced by quercetin in the cerebral cortex and hippocampus of APP/PS1 mice [170]. Other in vivo studies revealed that quercetin decreased extracellular β-amyloidosis, tauopathy, astrogliosis, and microgliosis in the hippocampus and amygdala of 3xTg-AD mice, decreasing the number of paired helical filaments (PHF), Aβ levels, and BACE1-mediated cleavage of APP [171,172]. In quercetin-treated 3xTg-AD mice, reactive microglia and Aβ aggregates were reduced [173], and the oral administration of quercetin increased brain apolipoprotein E (ApoE) and decreased Aβ levels in the cerebral cortex of 5xFAD mice model [174]. Moreover, increased Aβ clearance and decreased astrogliosis were observed in APP/PS1 mice receiving a quercetin-enriched diet during the early-middle stage of AD-like pathology progression [175]. In Aβ-injected mice, an animal model of sporadic AD, it was also demonstrated that protein levels of APP and BACE as well as of p-tau were reduced by quercetin [176]. Finally, it was recently reported that quercetin administration decreased the amount of Aβ in the hippocampal CA1 regions of Aβ-injected rats [177]. Additionally, the oral administration of rutin decreased oligomeric Aβ levels in brain of APP/PS1 transgenic mice [178].

### 3.2. Oxidative Stress

**Figure 4:**
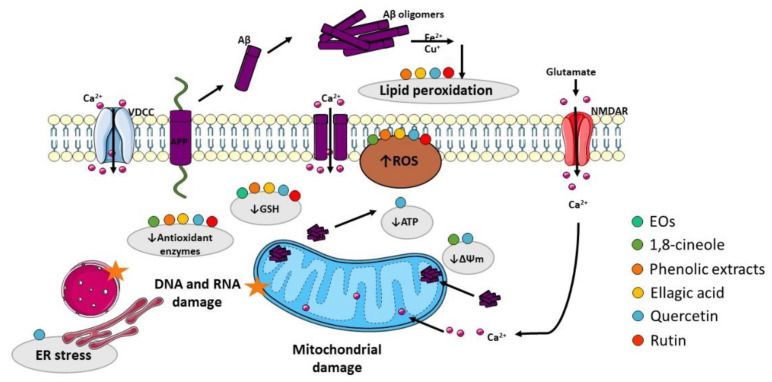
Effect of compounds obtained from *E. globulus* leaves in oxidative stress and mitochondrial damage in AD. Aβ oligomers can insert the plasma membrane originating pores by which Ca^2+^ pass into the cytoplasm. Aβ can also interact with metal ions (Fe^2+^ and Cu^+^) to generate reactive oxygen species (ROS), which cause membrane lipid peroxidation. As consequence, the membrane turns depolarized, and voltage-dependent Ca^2+^ channels (VDCC) and glutamate receptor-associated channels (in particular NMDAR, N-methyl-D-aspartate receptor) open increasing cytoplasmic Ca^2+^ content. Additionally, Aβ overproduction can cause mitochondrial damage, which culminates in ROS accumulation and ATP depletion that can impair axonal transport consequently originating abnormal mitochondrial dynamics and promoting neurotransmission deficits. ATP depletion can also lead to ionic alterations in the cytosol due to dysfunction of ATP-dependent ion channels. Moreover, ROS accumulation affects the mitochondrial permeability transition pore (MPTP), which further potentiates mitochondrial damage due to Ca^2+^ overload and inhibition of the electron transport chain. ROS increase also promotes damage to proteins, namely DNA and RNA.

The presence of oxidative stress markers in the AD brain has been pointed out as another relevant AD hallmark. Oxidative stress is caused by an imbalance between the production of reactive oxygen species (ROS) and/or reactive nitrogen species (RNS) and the removal capacity of the antioxidant system, promoting damage to lipids, proteins, ribonucleic acids (RNA), and deoxyribonucleic acids (DNA) [179,180]. Despite the mechanisms by which the redox balance is altered in AD and the sources of ROS/RNS remain unknown, numerous studies suggest that Aβ is a potent trigger of oxidative stress that is, at least in part, mediated by the disruption of mitochondrial function and subsequent generation of oxidant species [181] (Figure 4). Therefore, development of novel antioxidant strategies is required to prevent AD progression.

Many studies reported the antioxidant properties of EOs from *E. globulus*, which contribute to its neuroprotective effects [17,116]. For example, an in vitro study performed by Mizuno (2015) found that hydrogen peroxide (H_2_O_2_)-induced neuronal death was attenuated by the EO of *E. globulus* [182]. Moreover, Yadav (2019) showed that *E. globulus* oil alleviated depressive and cognitive symptoms of ketamine-induced psychosis in rats mediated by its antioxidant effect in the cerebral cortex and hippocampus, where the levels of reduced glutathione (GSH) were restored [77]. In both studies, the reported protective effect of EO from *E. globulus* might be due to the presence of 1,8-cineole, which was shown to be the major component. In fact, Ryu (2014) showed that 1,8-cineole may attenuate oxidative stress in cortical neuronal/glial cells through its antioxidant capacity as ROS scavenger and activator of superoxide dismutase (SOD) [183]. Additionally, an in vitro study using a neuronal cell model, performed by Khan and colleagues in 2014, demonstrated that Aβ-induced neuronal toxicity was prevented by 1,8-cineole pretreatment. The loss of mitochondrial membrane potential as well as ROS accumulation were attenuated by 1,8-cineole, supporting its anti-oxidative properties [184]. On the other hand, as observed above in the previous section, α-pinene is also present in the EO of *E. globulus* leaves and in vivo studies revealed its antioxidant effect. Lee (2017) demonstrated that α-pinene increased protein levels of antioxidant enzymes, namely the heme oxygenase-1 (HO-1) and manganese superoxide dismutase (MnSOD) in the hippocampus of the scopolamine-induced AD mice model via activation of the nuclear factor erythroid 2-related factor 2 (Nrf2) [185], which is a transcription factor that stimulates an antioxidant defense response. Nrf2 levels decrease with age, and reduced Nrf2 levels were reported in AD animal models and postmortem human brain tissue from patients [186]. Interestingly, recent studies revealed that Nrf2 activators may delay the progression and ameliorate the symptoms of the disease, suggesting that Nrf2 inducers might be relevant therapeutic molecules for AD [187].

González-Burgos (2018) investigated the antioxidant activity of different extracts (acetone, ethanol, and methanol) from *E. globulus* leaves and concluded that the extracts rich in phenolic compounds were effective to prevent H_2_O_2_-induced oxidative stress and preserve cell viability, increasing the activity of antioxidant enzymes and GSH levels as well as decreasing lipid peroxidation and ROS production in SH-SY5Y cells [13]. As mentioned before, the ellagic acid is one of the most predominant compounds found in phenolic extracts from *E. globulus* leaves. In fact, several studies reported the antioxidant properties of ellagic acid with significant impact on the progression of AD pathology, particularly through the activation of several antioxidant enzymes, reducing lipid peroxidation and free radical scavenging activity. Kabiraj and collaborators (2014) showed that ellagic acid is able to scavenge peroxynitrite, protecting PC12 cells against rotenone-induced cell death and also to reduce ROS and RNS production in these neuronal-like cells. Moreover, these authors demonstrated that ellagic acid suppressed apoptosis caused by rotenone by reducing poly (ADP-ribose) polymerase-1 (PARP) cleavage, which is a hallmark of apoptotic cell death [188]. Shen and co-authors (2017) also found that ellagic acid protected PC12 cells from Aβ-induced damage by inhibiting ROS production and reducing calcium ion influx [152]. Furthermore, ellagic acid pretreatment in intrahippocampal Aβ-microinjected rats, a model that mimics early-onset AD, mitigated oxidative stress by increasing the antioxidants catalase (CAT) and GSH and reducing the levels of malondialdehyde (MDA), a lipid peroxidation product [189]. Other study of Jha (2018) used STZ to induce a sporadic AD-like phenotype in rats and observed a decrease in oxidative stress profile after treatment with ellagic acid. Ellagic acid-treated animals revealed higher brain levels of mitochondrial ATPase and a marked dose-dependent free radical scavenging activity. In addition, this study reported attenuation of MDA levels together with an increase in GSH levels and activation of CAT in animals treated with STZ in the presence of ellagic acid [153]. Consistently, two other studies reported that ellagic acid administration reduced the production of thiobarbituric acid reactive substances (TBARS) and prevented the depletion of the antioxidant GSH and inhibition of SOD and CAT activities in STZ-treated rats [190,191]. Furthermore, an in vivo study performed by Uzar (2012) demonstrated that ellagic acid protected neurons against oxidative damage in STZ-induced diabetic rats, decreasing lipid peroxidation and total oxidant status and oxidative stress index. Additionally, ellagic acid attenuated the effects of STZ on activated CAT and paraoxanase-1 (PON-1) enzymes [192].

Quercetin and its glycoside rutin are two abundant compounds found in phenolic extracts of eucalyptus leaves, and several in vitro and in vivo studies have investigated their neuroprotective potential in AD. Both compounds were reported to attenuate oxidative stress in different AD models, mainly by decreasing ROS production and lipid peroxidation and increasing GSH content and the activity of several antioxidant enzymes. In APPswe cells, which are a cellular model of AD consisting of cells transfected with Swedish mutated human APP, Jimenez-Aliaga and collaborators (2011) demonstrated that quercetin and rutin decrease ROS generation and lipid peroxidation and increase intracellular GSH content, improving the redox status of APPswe cells treated with H_2_O_2_ [155]. In addition, rutin and quercetin were found to have free radical scavenging activity and to ameliorate Aβ-induced neuronal death in mouse primary cortical neuronal cultures [193]. Moreover, rutin attenuated mitochondrial damage and reduced the levels of ROS and oxidized glutathione (GSSG) as well as the formation of MDA and stimulated the activity of the antioxidant enzymes CAT, SOD, GSH, and glutathione peroxidase (GPx) in microglia cells exposed to Aβ [156]. Rutin was also demonstrated to inhibit amylin-induced neurotoxicity in SH-SY5Y cells, reducing the formation of ROS, GSSG, and MDA; attenuating mitochondrial damage and increasing the GSH/GSSG ratio; and enhancing the antioxidant activity of SOD, CAT, and GPx [194]. Additionally, quercetin was shown to preserve cell viability in PC12 cells treated with H_2_O_2_ [195]. An in vitro study with primary hippocampal cultures described that low doses of quercetin significantly attenuated Aβ-induced cytotoxicity, lipid peroxidation, protein oxidation, and apoptosis; however, higher dosages were reported to potentiate neuronal dysfunction [196]. Later studies demonstrated that quercetin protected rat primary hippocampal neurons against H_2_O_2_- or Aβ-induced neurotoxicity, attenuating ROS accumulation and depolarization of the mitochondrial membrane [197]. The role of quercetin in OA-induced oxidative stress in HT22 hippocampal cells was investigated, and it was found that pre-treatment with quercetin activates SOD, avoids GSH depletion, and decreases ROS production and MDA levels. The alterations in membrane potential caused by OA were reversed by quercetin, further supporting its neuroprotective action [164]. Quercetin was also reported to raise intracellular GSH content and prevent oxidative/nitrosative damage to DNA, lipids, and proteins in SH-SY5Y cells exposed to a neurotoxin [198]. On the other hand, rutin pretreatment was shown to decrease TBARS and PARP activity and increase GSH content and the activity of GPx, glutathione reductase, and CAT enzymes in the hippocampus of rats treated with STZ [199]. The effect of rutin was investigated in APPswe/PS1dE9 transgenic mice, and it was demonstrated that it decreased GSSG and MDA levels and increased SOD activity and GSH/GSSG ratio [178]. Moreover, lipid peroxidation was decreased in the brain, liver, and kidneys by treatment with rutin in an AD mouse model induced by Aβ injection [200]. Oxidative damage was attenuated by rutin treatment in rats with chronic cerebral hypoperfusion, namely GPx activity were increased, and the levels of MDA and protein carbonyls were decreased in rutin-treated animals [201]. Furthermore, it was recently demonstrated that pretreatment with rutin reduced CAT, GSH, and SOD protein levels in rats injected with doxorubicin [202]. Furthermore, an in vivo study performed by Tota and collaborators showed that quercetin restored cerebral blood flow and adenosine triphosphate (ATP) content after STZ administration in mice and reduced oxidative and nitrosative stress as demonstrated by a reduction in MDA and by an increase in GSH content [203]. It was also reported that quercetin treatment reduced MDA levels in the brain of STZ-induced diabetic rats [204]. In addition, quercetin decreased MDA generation in brain homogenates of mice treated with trimethyltin and showed strong antioxidant capacity determined through free radical scavenging activity assays [205]. Furthermore, lipid peroxidation was shown to be significantly inhibited by quercetin in the brain of Aβ-injected mice [206]. Indeed, increased SOD, CAT, and GSH and decreased MDA levels were observed in the brain of Aβ-injected rats treated with quercetin, concomitantly with activation of the antioxidant Nrf2/HO-1 pathway [177]. Quercetin ameliorated mitochondrial dysfunction, as evidenced by restoration of mitochondrial membrane potential and ROS and ATP levels in mitochondria isolated from the hippocampus of APP/PS1 transgenic mice. Furthermore, the activity of AMPK, which is a master regulator of cellular energy and metabolism, was significantly increased by quercetin [170]. Recent studies demonstrated that quercetin prevented the mitochondrial apoptotic pathway and neuronal degeneration by a mechanism involving regulation of BAX/Bcl2 and reduction of caspase-3 activity, cytochrome c release, and PARP cleavage in the brain of mice treated with lipopolysaccharide (LPS) [207]. Finally, reduction of MDA levels in animals injected with Aβ by rutin and quercetin were associated with upregulation of cAMP-response element binding protein (CREB) and brain-derived neurotrophic factor (BDNF) [208,209], which is an important regulator of neuronal growth and synaptic plasticity. CREB is one of the essential regulators of BDNF since its phosphorylated form binds to a specific sequence in the BDNF promoter and controls its transcription [210].

Several evidences support a crosstalk between oxidative stress and endoplasmic reticulum (ER) stress. In AD, the accumulation of misfolded proteins in susceptible brain regions suggests that the impairment of ER proteostasis machinery is involved in AD pathophysiology [211]. Therefore, ER stress can be considered as a therapeutic target for AD treatment. Under conditions of misfolded proteins overload within the ER lumen, ER stress sensors initiate the unfolded protein response (UPR) to reestablish homeostasis. This pathway comprises the activation of three ER trans-membrane proteins, namely inositol-requiring enzyme 1α (IRE1α), PKR-like ER kinase (PERK), and activating transcription factor 6 (ATF6) [210,212]. IRE1α activation promotes splicing of X-Box-binding protein 1 (XBP1)-mRNA [212] and the spliced XBP1 accumulated inside the nucleus upregulates crucial genes to reestablish global proteostasis under ER stress [213]. Furthermore, IRE1α can also activate relevant signaling mediators, namely c-Jun N-terminal kinase (JNK), which regulates autophagy and apoptosis [214]. ATF6 is an ER-membrane-bound transcription factor that triggers the transcription of ER molecular chaperones [210]. PERK also acts as an ER stress sensor, and under stress conditions, the α-subunit of eukaryotic initiation factor 2 (eIF2α) is oligomerized and phosphorylated by PERK [212]. This inhibits global protein translation, decreasing the overload of misfolded proteins [215,216]. Moreover, eIF2α phosphorylation increases translation of the activating transcription factor 4 (ATF4), which encodes genes of autophagy and proteins responsible for cell redox and metabolic regulation [216]. In addition, under chronic ER stress, ATF4 upregulates the transcription factor C/EBP homologous protein (CHOP), GADD34, and numerous members of Bcl2 family such as BAX and BAK, two central apoptotic regulators [217]. GADD34 can revert the eIF2α phosphorylation in a feed-forward cycle to close PERK signaling [218]. There are some evidences that quercetin ameliorates ER stress in AD models. In 2015, Hayakawa and colleagues reported that quercetin can rescue proteostasis, decreasing eIF2α phosphorylation, ATF4 expression, and Aβ secretion through GADD34 upregulation in cells upon autophagy impairment or ER stress conditions, which was confirmed in vivo using an AD mouse model [219]. In addition, quercetin repressed ER stress by reducing phosphorylation of eIF2α, PERK, and IRE1α; suppressed oxidative stress by reducing intracellular ROS production; and restored mitochondrial membrane potential in OA-treated SH-SY5Y cells. The same study also reported reduced IRE1α and PERK phosphorylation in mice exposed to high-fat diets [167]. A recent study performed by Woo and co-authors in Aβ-injected mice revealed that quercetin attenuates oxidative stress, namely ROS and TBARS generation. Under these conditions, a decrease was observed in the levels of ER stress markers such as phosphorylated eIF2α and PERK, XBP1, and CHOP as well as of pro-apoptotic Bax, phosphorylated JNK, and cleaved caspases-3 and -9 together with upregulation of the anti-apoptotic protein Bcl2 [176].

### 3.3. Inflammation

**Figure 5:**
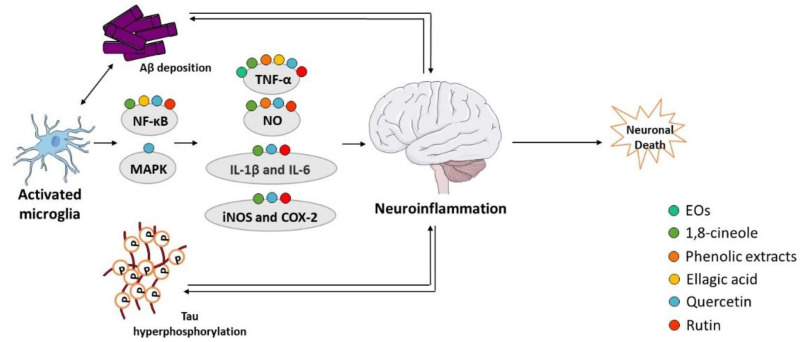
Effect of compounds obtained from *E. globulus* leaves on neuroinflammation in AD. A vicious circle between Aβ and tau accumulation in the brain, microglia activation, and release of pro-inflammatory cytokines culminates in neuronal death in AD.

Recent evidences suggest that inflammation has a fundamental role in AD pathogenesis; therefore, controlling the interactions between the nervous and the immune system might be crucial to prevent or delay the disease [220]. Brain inflammation seems to play a neuroprotective role in acute-phase responses but becomes deleterious during a chronic response to toxic insults [221]. Activated microglia release a diversity of proinflammatory and toxic products, including ROS, nitric oxide (NO), and cytokines such as interleukin-1β (IL-1β), interleukin-6 (IL-6), and tumor necrosis factor alpha (TNF-α), which play a significant role in the neuroinflammatory process. Aβ peptide increases the levels of cytokines, including TNF-α and IL-1β, and in turn, elevated levels of IL-1β potentiate Aβ accumulation [220,222]. Additionally, elevated levels of IL-1β can increase the production of other cytokines, such as IL-6, which activates the CDK-5 kinase that can lead to tau hyperphosphorylation [223]. Neuroinflammation has emerged as a third relevant hallmark in AD that can act as a link between amyloid and tau pathologies [224] (Figure 5). In fact, immune-related cells and proteins have been reported to be located within close proximity to senile plaques [225,226], and some evidences indicate that the prolonged use of nonsteroidal anti-inflammatory drugs (NSAIDs) reduce the risk to develop AD and delays the progression of the disease [227], possibly due to the inhibition of cyclooxygenases (COX) and activation of peroxisome proliferator-activated receptor γ (PPARγ) [227]. COX expression is repressed by NSAIDs, which declines the synthesis of prostaglandins and decreases the secretion of cytokines [228]. There are also evidences that NSAIDs decrease the level of Aβ in neuronal cell cultures and transgenic mice modelling AD [227]. Nevertheless, additional studies are required to confirm the beneficial effect of NSAIDs in AD.

*E. globulus* EO and its major component were recently reported to have anti-inflammatory activity relevant in the AD context. It has been previously reported that the expression of proinflammatory cytokines TNF-α, IL-1β, and IL-6 was lowered by 1,8-cineole in cells exposed to Aβ, and 1,8-cineole also succeeds in reducing NO accumulation and downregulating inducible NO synthase (iNOS), COX-2, and NF-κB [184]. More recently, the EO from *E. globulus* was demonstrated to reduce the serum levels of TNF-α in rats with psychosis, in the absence of any other significant alteration in inflammatory markers [77].

There are some evidences that extracts from eucalyptus leaves and ellagic acid reduce inflammation through depletion of TNF-α levels in AD models. Akhtar and collaborators extracted eucalyptus leaves with ethanol and detected anti-inflammatory activity, as shown by inhibition of TNF-α and NO production in macrophages exposed to LPS and interferon-γ (INF-γ) [229]. An in vitro study performed in cultured primary murine cortical microglia demonstrated that ellagic acid decreases Aβ-induced TNF-α secretion [230]. Another in vivo study showed that the reduction of hippocampal nuclear/cytoplasmatic Nrf2 ratio in Aβ-microinjected rats was reversed by ellagic acid treatment, which also reverted the alterations in NF-κB and TLR4 expression [189]. Moreover, ellagic acid was shown to prevent the accumulation of TNF-α detected in the STZ-induced AD rat model [190,191].

The anti-inflammatory effects of quercetin and rutin in AD models has been reported in several studies, which describe a decrease in NO production and in the expression of proinflammatory cytokines. Regarding in vitro studies, Wang and co-authors observed that rutin reduced NO formation and iNOS activity and also modulated the production of proinflammatory cytokines by decreasing TNF-α and IL-1β generation in microglia cells treated with Aβ [156]. Similarly, rutin was showed to reduce the production of NO, iNOS activity, and release of the pro-inflammatory cytokines TNF-α and IL-1β in amylin-treated SH-SY5Y cells, attenuating neurotoxicity [194]. Additionally, a study performed in LPS-stimulated microglia cells reported that rutin decreases expression levels of TNF- α, IL-1β, IL-6, and iNOS as well as the secretion of IL-6, TNF-α, and NO and increases the production of interleukin-10 (IL-10), the M2 regulatory cytokine, as well as arginase. Moreover, rutin also restored LPS-induced upregulation of COX-2, interleukin-18 (IL-18), and transforming growth factor-β (TGF-β) [231]. Similarly, in vitro studies have also linked quercetin’s neuroprotective effect with its anti-inflammatory activity. For example, quercetin was shown to prevent the release of TNF-α and IL-6 from activated microglia and astrocytes and attenuated the activation of proinflammatory signaling pathways such as MAPK and NF-κB [198]. Thioredoxin-interacting protein (TXNIP) is a crucial node in ER stress and NLR family pyrin domain containing 3 (NLRP3) inflammasome, which activates caspase-1, leading to IL-1β secretion to cause inflammation in cells or tissues [232]. NLRP3 inflammasome is a protein complex that comprises NLRP3, the adaptor protein apoptosis-associated speck-like protein containing a C-terminal caspase-activation-and-recruitment domain (CARD) (ASC), and the precursor pro-caspase-1. Consistent with this, quercetin suppressed TXNIP expression and NLRP3 inflammasome activation indicated by downregulation of NLRP3, ASC, and procaspase-1 in OA-treated SH-SY5Y cells. Quercetin effectively reduced IL-1β and IL-6 production in neuronal cells and restored NLRP3 activity and reduced IL-1β and TNF-α production in mice exposed to a high-fat diet [167]. Quercetin also attenuated neuroinflammation in a mouse model of AD decreasing IL-1β and monocyte chemoattractant protein-1 (MCP-1) levels [233]. A study using quercetin-treated 3xTg-AD mice showed a reduction in reactive microglia and astrocytes, glial fibrillary acidic protein (GFAP), iNOS, and COX-2 immunoreactivity as well as IL-1β levels in hippocampal lysates [173]. Quercetin also reduced LPS-induced gliosis and the levels of various inflammatory markers, such as TNF-α, COX-2, and iNOS, in the cortex and hippocampus of adult mice [207]. Finally, quercetin decreased NO formation in STZ and Aβ- injected mice [203,206]. In vivo studies with rutin also disclosed its anti-inflammatory activity in AD context. Indeed, rutin ameliorated STZ-induced inflammation in rats by decreasing NO levels and the expression of GFAP, interleukin-8 (IL-8), COX-2, iNOS, and NF-κB [199]. Rutin also inhibited glial activation; reduced the levels of pro-inflammatory cytokines TNF-α, IL-1β, and IL-6; and prevented neuronal damage in rats with chronic cerebral hypoperfusion [201]. Other study showed that chronic treatment with rutin decreases TNF-α levels in the hippocampus and frontal cortex of rats injected with doxorubicin [202]. The oral administration of rutin was also found to downregulate microgliosis and astrocytosis and to reduce IL-1β and IL-6 levels in the brain of the APP/PS1 transgenic AD mice model [178]. Additionally, the NO formation was reduced by rutin in Aβ-injected mice [200].

### 3.4. Cholinesterase Activity

**Figure 6:**
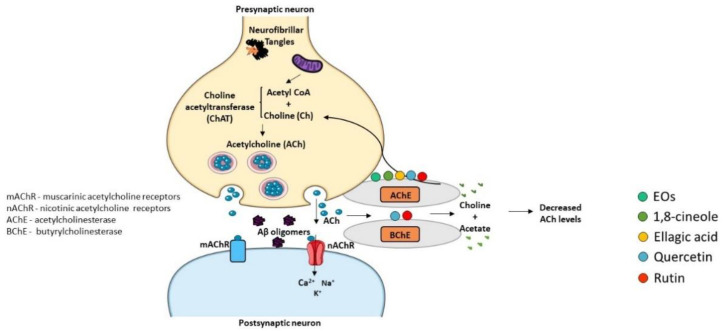
Effect of compounds obtained from *E. globulus* leaves on cholinesterase activity in AD. Synthesis of acetylcholine (ACh) neurotransmitter from acetyl coenzyme A (Acetyl CoA) and choline (Ch) occurs by the action of the enzyme choline acetyltransferase (ChAT) in the presynaptic terminal. Acetylcholine is released in the synaptic cleft, where it can activate both muscarinic (mAChR) and nicotinic (nAChR) receptors. Acetylcholinesterase (AChE) or butyrylcholinesterase (BChE) break acetylcholine into choline and acetate. ACh levels are low in AD brains and cholinergic neurotransmission in impaired. AChE and BChE inhibitors correct these deficits increasing the amount of ACh that remains in the synaptic cleft and interacts with postsynaptic receptors.

The “Cholinergic Hypothesis” is central to explain AD pathophysiology (Figure 6). This hypothesis considers that cholinergic neurons are affected in AD, leading to a decrease in the synthesis of the neurotransmitter ACh and subsequent release to the synaptic cleft, resulting in cognitive decline and memory loss [234,235]. Therefore, inhibitors of AChE and butyrylcholinesterase (BChE) enzymes that degrade ACh can represent a therapeutic strategy to increase the levels of ACh in the synaptic cleft and its binding to post-synaptic receptors, thus potentiating cholinergic neurotransmission. In fact, three of the four drugs approved for the relief of AD symptoms are AChE inhibitors, namely donepezil, rivastigmine, and galantamine [5]. The active sites of AChE/BChE enzymes bind these cholinesterase inhibitors in a reversible manner and avoid ACh degradation, facilitating cholinergic neurotransmission. Thus, AD symptoms are ameliorated due to the rise of ACh concentration in the synaptic cleft [236]. However, the efficacy of cholinesterase inhibitors in AD treatment is limited, and side effects have been reported, such as nausea, abdominal pain, diarrhea, dyspepsia, vomiting, and skin rash [237]. Hence, the discovery of new cholinesterase inhibitors from medicinal plant sources concomitantly presenting less adverse effects can be a valuable strategy.

The effective in vitro inhibition of AChE activity by *E. globulus* EO has been described [16]. Moreover, studies in cellular models detected anti-cholinesterase activity of 1,8-cineole and α-pinene [238,239]. In addition, the AChE inhibitory activity of eucalyptus EO in the hippocampus region of rat’s brain with psychotic symptoms was recently reported [77]. Additionally, mRNA levels of enzymes involved in ACh metabolism were evaluated in the cortex of scopolamine-induced amnesic animals, and it was observed that the administration of α-pinene reverted the decrease in the mRNA levels of choline acetyltransferase (ChAT), which is responsible for the formation of ACh [185]. However, mRNA levels of AChE were not altered by scopolamine treatment in the presence or absence of α-pinene. These studies revealed the neuroprotective potential of *E. globulus* EO and its major compounds due to their capacity to inhibit AChE activity.

Ellagic acid was recently described to reduce AChE activity in the brain of animals injected with Aβ [189] or with STZ [153,190], supporting the ability of ellagic acid to reduce cerebral ACh degradation and its neuroprotective role.

Quercetin and rutin also demonstrated to inhibit AChE activity, revealing neuroprotective effects, particularly in AD. In fact, a study of Ademosun and colleagues showed that both compounds significantly decrease AChE and BChE activities in rat brain homogenates, but quercetin showed a higher inhibitory ability than rutin [240]. One docking study concluded that rutin exhibited an elevated docking score against AChE in comparison with quercetin, suggesting that rutin is a promising drug candidate for AD [241]. Rutin treatment was also found to alleviate ACh depletion and ChAT inhibition as well as the activation of AChE caused by cerebral hypoperfusion in rats [201]. On the other hand, in vitro studies demonstrated that quercetin has a strong inhibitory effect against AChE and BChE enzymes [160,242,243], and a relevant role of quercetin as an AChE inhibitor has been described, supporting its therapeutic potential for AD [244,245,246]. Accordingly, several other in vitro studies found similar or higher AchE inhibitory activity of quercetin over conventional AchE inhibitors [247]. It was observed that quercetin has significant AChE inhibitory activity almost similar to that of huperzine A [248] or donepezil [249], which are well-known AChE inhibitors. In addition, these results were confirmed in vivo, and quercetin has been reported to attenuate the AChE activity in the brain of STZ-treated mice [203,204]. Another study revealed that quercetin suppressed AChE activation in a dose-dependent manner in brain tissues of mice exposed to neurotoxic trimethyltin [205]. Finally, Liu and co-authors showed that quercetin was able to restore cortical ACh levels and inhibit AChE activity in Aβ-injected mice [209].

### 3.5. GABAergic and Glutamatergic Dysfunction

**Figure 7:**
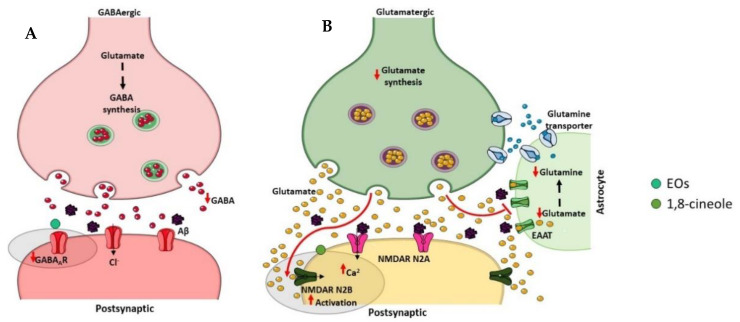
Effect of compounds obtained from *E. globulus* leaves on inhibitory (**A**) and excitatory (**B**) synapses in AD. (**A**) The inhibitory γ-aminobutyric acid (GABA) synapse. GABA is synthesized from glutamate by the glutamic acid decarboxylase enzymes in the presynaptic terminal of GABAergic neuron. The vesicular GABA transporter packs GABA into vesicles, which, after release in the synaptic cleft, binds GABA_A_ receptors localized on the postsynaptic neuron. The reuptake of GABA into the presynaptic axon stops the GABA action in the synapse. GABA levels are significantly reduced in AD patients as well as the GABA_A_ receptor density. (**B**) The excitatory glutamate synapse. Glutamine is converted to glutamate via glutaminase in the presynaptic terminal of glutamatergic neuron, and the vesicular glutamate transporter packs glutamate into vesicles. After glutamate release in the synaptic cleft, it acts on glutamate receptors localized on the postsynaptic neuron. The excitatory amino acid transporters (EAATs) present in nearby astrocytes clear the glutamate from the synaptic cleft. Glutamate is converted to glutamine via glutamine synthetase in astrocytes before being transported to presynaptic neurons. In AD, Aβ oligomers affect extrasynaptic N-methyl-D-aspartate (NMDA) receptors enriched in NR2B subunits, leading to an excessive activation and consequently to an excess of Ca^2+^ accumulation in the post-synaptic cell.

γ-Aminobutyric acid (GABA) is a major inhibitory neurotransmitter in the human brain, which plays a relevant role in cognitive functions [250] (Figure 7A). Significant reductions in cerebral GABA levels have been described in AD patients as well as in AD animal models [251]. GABA_A_ is one isoform of GABA receptors, and some studies have demonstrated decreased GABA_A_/benzodiazepine (BZD) receptor density [252,253] and expression levels [254] in the brain of AD patients. Interestingly, the role of selective GABA_A_ agonists to counteract Aβ-induced toxicity was showed [255] suggesting that the GABAergic system is involved in the pathophysiology of AD and therefore may be a potential therapeutic target for this neurodegenerative disorder. Recently, it was found that eucalyptus oil increases brain GABA levels [77], and α-pinene acts as a partial modulator of GABA_A_-BZD receptors and binds directly to the BZD binding site of the GABA_A_ receptor [256].

Glutamate is an excitatory neurotransmitter typically present in the hippocampus and cerebral cortex that plays an important role in learning and memory [257] (Figure 7B). There are two types of post-synaptic glutamate receptors, ionotropic and metabotropic G protein-coupled receptors, which modulate calcium and sodium influx into neuronal cells [258]. However, excessive activation of glutamate receptors, in particular the N-methyl-D-aspartate (NMDA) subtype of ionotropic receptors, provokes excitotoxic neuronal death [259]. In AD, an excessive activation of the NMDA receptor has been described and contributes to the neurodegenerative process in consequence of the excessive influx of calcium [259]. Numerous evidences suggest that blocking excitotoxicity might be beneficial in AD. Indeed, memantine, which was approved by FDA and EMA for the treatment of AD symptoms, is an uncompetitive NMDA receptor antagonist that blocks excitoxicity with minimal side effects due to the preservation of normal glutamatergic transmission [260]. A study using computational models proposed 1,8-cineole as a good candidate for NMDA antagonism comparing its molecular features with the conventional ligand memantine [261].

These promising findings suggest that the effect of *E. globulus* EO on GABAergic and glutamatergic transmission should also be explored as therapeutic strategies for AD. Nevertheless, there is no information in the literature about phenolic compounds and AD-associated perturbation of GABAergic and glutamatergic neurotransmission.

### 3.6. Impaired Learning and Memory

**Figure 8:**
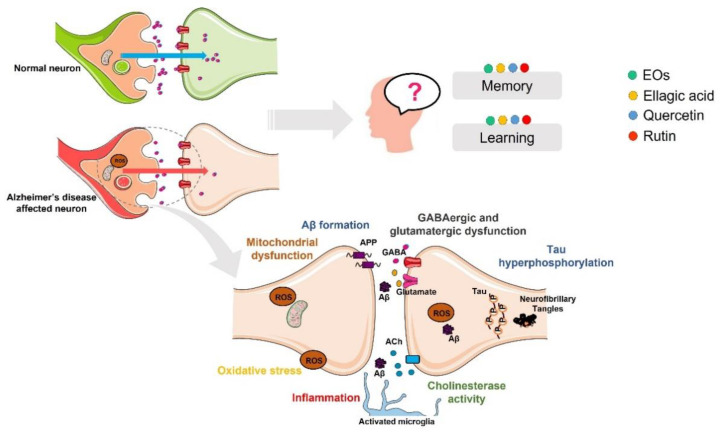
Effect of compounds obtained from *E. globulus* leaves in the AD-associated memory and learning impairment.

Learning is the process of acquiring new information, while memory is the process of storing this information to use it for future purposes (Figure 8). Cognition is defined as the combination of learning and memory and is strictly dependent on the concerted action of several neurotransmitters.

In AD, the stage and severity of the disease are determined by the compromise in cognition [17]. The deterioration of cholinergic neurons have been reported to be implicated in cognitive deficits in AD patients [262]. Accordingly, anticholinergic agents such as scopolamine have been reported to induce memory deficits [263], and on the other hand, an improvement of the cholinergic system can revert alterations in cognition [264]. Based on this, and as previously stated, AChE and BChE inhibitors demonstrated to revert cognitive symptoms and have been approved for AD treatment.

EO from *E. globulus* was recently demonstrated to be able to restore learning and memory function in rats treated with ketamine that induces psychosis [77]. In addition, the administration of α-pinene attenuated learning and memory impairments induced in rats treated with scopolamine [185].

Several in vivo studies also support the beneficial effects of ellagic acid in cognition of AD animal models. For example, it was demonstrated that ellagic acid efficiently prevents scopolamine- and diazepam-induced cognitive impairments without affecting animals’ locomotion [265]. Treatment with ellagic acid also showed to ameliorate memory and spatial learning alterations in the APP/PS1 transgenic AD mice model [154]. Moreover, the study of Kiasalari and collaborators described that ellagic acid ameliorates learning and memory performance in Aβ-injected rats [189]. Regarding the STZ-induced AD rat model, it was observed that ellagic acid prevents the STZ-induced cognitive deficits in animals without affecting locomotor activity and motor coordination [153,190,191].

Strong evidences support that quercetin and rutin prevent cognitive impairments in several AD animal models. Pretreatment with quercetin and rutin prevented scopolamine-induced memory impairment in zebrafish without locomotor alterations [266]. Rutin also ameliorated deficits in learning and memory in STZ rats [199] as well as the problems in spatial learning and memory, in working memory, and also in contextual memory in rats with chronic cerebral hypoperfusion [201]. In AD transgenic mice, it was demonstrated that rutin decreased spatial memory deficits [178] and alleviated cognition and memory impairments in Aβ-injected mice [200]. Furthermore, rutin restored short- and long-term episodic memory in scopolamine- and doxorubicin-treated rats without interfering with the locomotor activity of the animals [202,267]. Quercetin administration in aged and LPS-treated mice also enhanced the memory capacity in the absence of alterations in locomotion [207,268]. Furthermore, quercetin avoided STZ-induced memory impairment in mice [203] and enhanced spatial memory in rats [269]. Quercetin also prevented the impairment of memory and the anxiogenic-like behavior induced in STZ-diabetic rats [204]. Additionally, quercetin treatment attenuated trimethyltin-induced memory impairment in mice [205]. Moreover, in a study with mice exposed to a high-fat diet, quercetin administration enhanced cognition [167]. Another study also showed that a quercetin-enriched diet during the early-middle pathology stages ameliorated cognitive dysfunction in APP/PS1 mice [175]. In addition, beneficial effects of quercetin in learning, memory deficits, and cognitive function were demonstrated in APP/PS1, APP23, and 3xTg-AD transgenic mice models of AD [170,171,172,219]. Furthermore, quercetin administration in Aβ-induced amnesic mice enhanced learning and memory performance [206,209,270]. Finally, two studies with rats injected with Aβ also demonstrated the capacity of quercetin to enhance learning and memory [177,271]. Importantly, in early-stage AD patients, memory recall assessed using the Revised Hasegawa Dementia Scale was demonstrated to be enhanced by the intake of quercetin [272].

**Table 3:** Neuroprotective effects of EO, phenolic extracts, and the major constituents obtained from *E. globulus* leaves against various neurodegeneration model systems.

Compound	Model	Dose and Duration	Effects	Reference
EO	Cell free	IC_50_ = 0.1298 mg/mL	Inhibited AChE activity	[16]
	GT1-7 cells treated with H_2_O_2_	25 ppm, 24 h	Attenuated neuronal death	[182]
	Wistar albino rats treated with ketamine	500 and 1000 mg/kg/day, p.o., 21 days	Facilitated GABA release, increased GSH levels, inhibited dopamine neurotransmission, decreased TNF-α levels, and diminished AChE activity Restored learning and memory function	[77]
	Computational	-	Candidate for NMDA antagonism	[261]
Cineol	Cell free	IC_50_ = 840 μM	Inhibited AChE activity	[239]
	Differentiated PC12 cells treated with Aβ25-35	2.5, 5 and 10 μM, 24 h	Restored cell viability Reduced mitochondria membrane potential and ROS and NO levels Lowered expression of TNF-α, IL-1β, IL-6, iNOS, COX-2, and NF-κB	[184]
	Primary rat cortical neurons/glial	10 μM, 4 h	Increased SOD activity and reduced ROS production	[183]
α-pinene	Computational	-	Partially modulated GABA_A_-BZD receptors Directly bound to the BZD binding site of GABA_A_ receptor	[256]
	Brain slices	10 µM		
	C57BL/6N mice treated with pentobarbital	100 mg/kg, p.o.		
	C57BL/6 mice treated with scopolamine	10 mg/kg, i.p.	Improved cognitive dysfunction Increased expression of ChAT Increased protein levels of antioxidant enzymes Activated Nrf2	[185]
Phenolic extracts	SH-SY5Y cells treated with H_2_O_2_	5, 10, 25 and 50 µg/mL, 24 h	Increased cell viability, GSH levels, and antioxidant enzymes activity Decreased ROS production and lipid peroxidation levels	[13]
	RAW264.7 cells treated with LPS and INF-γ	51 and 83 µg/mL, 24 h	Inhibited NO and TNF-α production	[229]
Ellagic acid	Cell free	IC_50_ = 39 μM	Inhibited BACE-1 activity	[150]
	SH-SY5Y cells treated with Aβ1-42	5 and 10 μM, 48 h	Promoted oligomers loss Prevented neuronal death	[151]
	PC12 cells treated with Aβ25-35	0.5, 2.5, and 5 µM, 12 h	Attenuated Aβ-induced toxicity Inhibited ROS production and reduced calcium ion influx	
	PC12 cells treated with rotenone	10 µM, 24 h	Attenuated cell death Reduced ROS and RNS production Suppressed apoptosis	[188]
	Primary murine cortical microglia treated with Aβ1-42	10 µM, 24 h	Decreased TNF-α secretion	[230]
	APP/PS1 transgenic mice	50 mg/kg/day, i.g., 60 days	Ameliorated learning and memory deficits Reduced neuronal apoptosis and amyloid deposition Inhibited tau hyperphosphorylation and decreased GSK-3β activity	[154]
	Wistar rats treated with Aβ25-35	50 and 100 mg/kg/day, i.p., 7 days	Improved learning and memory deficits Mitigated oxidative stress by increasing CAT and GSH and reducing MDA levels Reduced AChE activity Modulated NF-κB/Nrf2/TLR4 signaling pathway	[189]
	Wistar rats treated with STZ	50 mg/kg/day, p.o., 30 days	Decreased brain Aβ levels Revealed marked dose-dependent free radical scavenging effect and higher BMA levels Reduced AChE activity Prevented cognitive disfunction	[153]
	Wistar rats treated with STZ	35 mg/kg/day, p.o., 4 weeks	Reduced TBARS production and prevented the depletion of GSH and the inhibition of SOD and CAT activities Increased TNF-α levels Reduced AChE activity Restored memory deficits	[190]
	Wistar rats treated with STZ	17.5 and 35 mg/kg/day, p.o., 28 days	Reduced TBARS production and prevented the depletion of GSH Increased TNF-α levels Restored memory deficits	[191]
	Diabetic rats treated with STZ	50 mg/kg/day, p.o., 21 days	Decreased lipid peroxidation and oxidative stress index Increased antioxidant enzymes Attenuated NO production	[192]
	Wistar rats treated with scopolamine and diazepam	30 and 100 mg/kg/day, i.p., 10 days	Prevented cognitive deficits	[265]
Quercetin	Computational	-	Candidate as AChE inhibitor	[247,249]
	Cell free	50 µM	Inhibited and destabilized Aβ fibril formation	[158]
	Cell free	1 mg/mL (76.2% and 46.8% inhibition)	Inhibited AChE and BChE activities	[242]
	Cell free	IC_50_ = 181 µM and IC_50_ = 203 µM	Inhibited AChE and BChE activities	[240]
	Cell free	IC_50_ = 354 µM and IC_50_ = 421 µM	Inhibited AChE and BChE activities	[243]
	Cell free	IC_50_ = 19.8 µM	Inhibited AChE activity	[244]
	Cell free	IC_50_ = 3.6 µM	Inhibited AChE activity	[245]
	Cell free	IC_50_ = 14.4 µM	Inhibited AChE activity	[246]
	Cell free	IC_50_ = 51 µM	Inhibited AchE activity	[248]
	Cell free Bacterial cells	IC_50_ = 124.6 μM	Decreased Aβ aggregation	[163]
	Cell free	-	Inhibited Aβ fibril formation	[157]
	HT22 cells treated with Aβ25-35	-	Attenuated neuronal death	
	HT22 cells treated with OA	5 and 10 µM, 12 h	Attenuated neuronal death Decreased levels of SOD, mitochondria membrane potential, GPx, MDA, and ROS Inhibited hyperphosphorylation of tau protein Inhibited apoptosis via the reduction of Bax and up-regulation of cleaved caspase 3 via the inhibition of PI3K/Akt/GSK-3β, MAPKs, and activation of NF-κB	[164]
	HT22 cells treated with OA	5 and 10 µM, 12 h	Attenuated tau protein hyperphosphorylation Inhibited the activity of CD-K5 Attenuated intracellular calcium rise	[165]
	Differentiated SH-SY5Y cells treated with OA	100 nM, 6 h	Decreased tau phosphorylation levels	[166]
	SH-SY5Y cells treated with OA	10 µM, 6 h	Suppressed ER stress with decreased phosphorylation of IRE1α and PERK Decreased ROS production and restored mitochondria membrane potential Inhibited TXNIP and NLRP3 inflammasome activation and downregulated ASC and pro-caspase-1	[167]
	C57BL/6J mice exposed to high-fat diets	50 mg/kg/day, p.o., 10 weeks	Reduced IL-1β and IL-6 production Attenuated tau phosphorylation Reduced IL-1β and TNF-α production Enhanced AMPK activity Inhibited IRE1α and PERK phosphorylation, NLRP3 expression, and tau phosphorylation Improved cognitive disorder	
	Cell free	1, 5 and 10 µM	Inhibited the formation of Aβ fibrils and disaggregated Aβ fibrils	[155]
	APPswe-transfected SH-SY5Y cells	25, 50, and 100 nM, 24 h	Decreased ROS production and lipid peroxidation Increased GSH content and the redox status	
	Cell free	IC_50_ = 55 μM and IC_50_ = 19 µM IC_50_ = 0.55 µM	Inhibited AChE and BChE activities Inhibited BACE activity	[160]
	SH-SY5Y cells treated with L-DOPA	10, 50, 250, and 1000 µM, 24 h	Attenuated neuronal death	
	7W CHO cells overexpressing APP	10, 25, and 50 µM, 24 h	Inhibited Aβ and sAPPβ production Regulated BACE expression	[162]
	SH-SY5Y cells treated with TNF-α	20 µM, 30 min		
	SH-SY5Y, U373, and THP-1 cells treated with LPS and INF-γ or INF-γ	33 µM, 8 h	Reduced oxidative/nitrative damage to DNA, lipids, and proteins Increased intracellular GSH content Reduced the release of TNF-α and IL-6 Attenuated the activation of MAPK and NF-kB	[198]
	PC12 cells treated with H_2_O_2_	10, 30, 60 and 100 µM, 2 h	Preserved cell viability	[195]
	Cell free	IC_50_ = 5.4 μM	Inhibited BACE activity	[161]
	Primary rat E18 cortical neurons	20 μM, 24 h	Decreased Aβ levels	
	Primary rat hippocampal neurons treated with Aβ1-42	5 and 10 μM, 24 h	Attenuated neuronal death, protein oxidation, lipid peroxidation, and apoptosis	[196]
	Primary rat hippocampal neurons treated with Aβ1-42 and H_2_O_2_	10 μM, 24 h	Attenuated neuronal death, ROS accumulation, and depolarization of mitochondrial membrane	[197]
	Primary mouse cortical neurons treated with Aβ25-35	30 μM, 24 and 48 h	Demonstrated free radical scavenging activity Ameliorated neuronal death	[193]
	Cell free	250 µM	Inhibited Aβ fibrilization	[168]
	*C. elegans* treated with Aβ1-42	73 µM, ~12 days	Increased % of survival	
	*C. elegans* treated with Aβ1-42	100 µM, 48 h	Increased proteasomal activity Enhanced the flow of proteins through the macroautophagy pathway	[169]
	Zebrafish treated with scopolamine	50 mg/kg/single dose, i.p.	Attenuated memory deficits	[266]
	APP/PS1 transgenic mice	20 and 40 mg/kg/day, 16 weeks	Improved cognitive deficits Reduced scattered senile plaques Ameliorated mitochondrial dysfunction by restoration of mitochondrial membrane potential, ROS, and ATP levels Increased AMPK activity	[170]
	APP/PS1 transgenic mice	2 mg/g diet, 12 months	Increased Aβ clearance and reduced astrogliosis Ameliorated cognitive dysfunction	[175]
	APP/PS1 transgenic mice	1% in mouse chow, 10 months	Attenuated neuroinflammation by reducing IL-1β and MCP-1 levels	[233]
	APP23 transgenic mice	0.5% in mouse chow, 52 weeks	Reduced eIF2α phosphorylation and ATF4 expression through GADD34 induction Improved memory deficits	[219]
	3xTg-AD mice	25 mg/kg/48 h, i.p., 3 months	Decreased extracellular β-amyloidosis, tauopathy, astrogliosis, and microgliosis Reduced PHF and Aβ levels Decreased BACE-1-mediated cleavage of APP Improved performance on learning and spatial memory	[171]
	3xTg-AD mice	100 mg/kg/48 h, p.o., 12 months	Reduced β-amyloidosis and tauopathy Improved cognitive deficits	[172]
	3xTg-AD mice	25 mg/kg/48 h, i.p., 3 months	Decreased reactive microglia and Aβ Reduced GFAP, iNOS, COX-2, and IL-1β	[173]
	5xFAD mice	500 mg/kg/day, oral gavage, 10 days	Increased brain ApoE and reduced Aβ levels	[174]
	ICR mice injected with Aβ1-42	50 and 100 mg/kg/day, p.o., 1 month	Improved learning and memory loss	[270]
	ICR mice injected with Aβ25-35	50 mg/kg/day, p.o., 2 weeks	Decreased protein levels of APP, BACE, and p-tau Reduced oxidative stress such as ROS and TBARS levels Decreased the protein levels of ER stress markers GRP78, p-PERK, p-eIF2α, XBP1, and CHOP and the proapoptotic molecules Bax, p-JNK, and cleaved caspases-3 and -9	[176]
	Mice injected with Aβ25-35	30 mg/kg/day, p.o., 14 days	Decreased NO formation and lipid peroxidation Improved cognitive function	[206]
	Kunming mice injected with Aβ25-35	5, 10, 20 and 40 mg/kg/day, oral gavage, 8 days	Regulated ERK/CREB/BDNF pathway Restored ACh levels and inhibited AChE activity Improved the learning and memory capabilities	[209]
	ICR mice treated with trimethyltin	5, 10, and 20 mg/kg/day, 3 weeks	Decreased MDA generation and showed antioxidant capacity Inhibited AChE activity Improved cognitive deficits	[205]
	C57BL/6N mice treated with LPS	30 mg/kg/day, i.p., 2 weeks	Prevented the mitochondrial apoptotic pathway and neuronal degeneration by regulating Bax/Bcl2, decreasing activated cytochrome c and caspase-3 activity, and cleaving PARP-1 Reduced activated gliosis and levels of various inflammatory markers such as TNF-α, COX-2, and iNOS Improved memory performance	[207]
	Swiss mice treated with LPS	25, 50, and 100 mg/kg/day, i.p., 7 days	Reversed memory deficits	[268]
	Sprague–Dawley rats injected with Aβ1-42	100 mg/kg/day, p.o., 19 days	Reduced Aβ levels Increased SOD, CAT, and GSH and decreased MDA levels Increased Nrf2/HO-1 pathway Improved cognitive deficits	[177]
	Wistar rats injected with Aβ1-42	40 mg/kg/day, p.o., 1 month	Alleviated learning and memory deficits	[271]
	Wistar rats treated with STZ	5, 25, and 50 mg/kg/day, oral gavage, 40 days	Reduced MDA levels Prevented the increase in AChE activity Prevented memory deficits	[204]
	Wistar rats treated with STZ	40 and 80 mg/kg/day, i.p., 12 days	Enhanced spatial memory	[269]
	Human early-stage AD patients	80 mg/patient/day, p.o., 4 weeks	Enhanced memory recall	[272]
Rutin	Computational	-	Candidate as AChE inhibitor	[241]
	Cell free	10 µM	Inhibited BACE activity	[159]
	Cell free	IC_50_ = 0.219 mM and IC_50_ = 0.288 mM	Inhibited AChE and BChE activities	[240]
	Cell free APPswe-transfected SH-SY5Y cells	1, 5, and 10 µM 100 µM 25, 50, and 100 nM, 24 h	Inhibited the formation of Aβ fibrils and disaggregated Aβ fibrils Inhibited BACE activity Decreased ROS production and lipid peroxidation Increased GSH content and the redox status	[155]
	Cell free	50 and 200 µM	Inhibited Aβ fibrillization and attenuated Aβ-induced cytotoxicity	[156]
	SH-SY5Y and BV-2 cells treated with Aβ1-42	0.8 and 8 µM, 24 h	Decreased ROS, NO, GSSG, and MDA formation Reduced iNOS activity and attenuated mitochondrial damage Increased GSH/GSSG ratio Enhanced SOD, CAT, and GPx activities Decreased TNF-α and IL-1β generation	
	Cell free SH-SY5Y cells treated with L-DOPA	IC_50_ = 3.8 nM 10, 50, 250, and 1000 µM, 24 h	Inhibited BACE activity Attenuated neuronal death	[160]
	SH-SY5Y cells treated with amylin	0.8, 4, and 8 µM, 24 and 48 h	Attenuated neuronal death Decreased the production of ROS, NO, GSSG, MDA, and pro-inflammatory cytokines TNF-α and IL-1β Attenuated mitochondrial damage and increased the GSH/GSSG ratio Enhanced the antioxidant enzyme activity of SOD, CAT, and GPx Reduced iNOS activity	[194]
	Primary mouse cortical neurons treated with Aβ25-35	30 μM, 24 and 48 h	Demonstrated free radical scavenging activity Ameliorated neuronal death	[193]
	Primary rat microglia treated with LPS	50 mM, 24 h	Decreased expression levels of TNF- α, IL-1β, IL-6, and iNOS Reduced the production of IL-6, TNF-α, and NO Increased production of the M2 regulatory cytokine IL-10 and arginase Restored upregulation of COX-2, IL-18, and TGF-β	[231]
	Zebrafish treated with scopolamine	50 mg/kg/single dose, i.p.	Attenuated memory deficits	[266]
	APP/PS1 transgenic mice	100 mg/kg/day, p.o., 6 weeks	Decreased oligomeric Aβ level Increased SOD activity and GSH/GSSG ratio Reduced GSSG and MDA levels Downregulated microgliosis and astrocytosis Decreased IL-1β and IL-6 levels Attenuated memory deficits	[178]
	ICR mice injected with Aβ25-35	100 mg/kg/day, p.o., 14 days	Decreased NO formation and lipid peroxidation Attenuated cognitive deficits	[200]
	Swiss albino mice treated with STZ	2.5, 5, and 10 mg/kg/day, p.o., 21 days	Restored cerebral blood flow and ATP content Reduced MDA and NO levels and increased GSH content Attenuated elevated AChE activity Prevented memory impairment	[203]
	Wistar rats injected with Aβ1-42	100 mg/kg/day, i.p., 3 weeks	Increased ERK, CREB, and BDNF expression and decreased MDA level Improved memory deficits	[208]
	Wistar rats treated with STZ	25 mg/kg/day, p.o., 3 weeks	Decreased TBARS, PARP activity, and NO level Increased GSH content and activities of GPx, glutathione reductase, and CAT Reduced the expression of COX-2, GFAP, IL-8, iNOS, and NF-kB Improved cognitive deficits	[199]
	Sprague–Dawley rats with chronic cerebral hypoperfusion	50 mg/kg/day, i.p., 12 weeks	Attenuated oxidative damage, namely increased GPx activity and decreased MDA levels and protein carbonyls Inhibited glial activation; reduced the levels of TNF-α, IL-1β, and IL-6; and prevented neuronal damage Alleviated ACh depletion, ChAT inhibition, and AChE activation Improved cognitive deficits	[201]
	Wistar rats injected with doxorubicin	50 mg/kg/day, p.o., 50 days	Reduced CAT, GSH, SOD, and TNF-α levels Prevented memory deficits	[202]
	Wistar rats treated with scopolamine	50 and 100 mg/kg/day, p.o., 15 days	Improved short- and long-term episodic memory deficits	[267]

## 4. Conclusions

Several efforts have been made to develop alternatives to the current therapies for AD treatment, which only alleviate symptoms without altering the progression of the disease, increasing efficiency, and decreasing side effects. A promising strategy to identify novel disease-modifying therapies is to test compounds extracted from natural resources. The present review discusses findings obtained in in vitro and in vivo studies performed with EO, phenolic extracts, and the major constituents obtained from *E. globulus* leaves in what concerns therapeutic effect and mechanism of action. Overall, 1,8-cineole was found to be the major compound present in EO, and ellagic acid, quercetin, and rutin are the main components of phenolic extracts from *E. globulus*, which were demonstrated to efficiently prevent or attenuate several AD-related hallmarks, namely amyloid and tau pathologies, oxidative stress and neuroinflammation, neurotransmission deficits, and also memory and learning impairments. The information reviewed herein suggests that extracts from *E. globulus* leaves could be used as raw material to develop efficient and safe nutraceuticals and/or plant-based medicinal products useful for AD prevention and novel therapies able to modify the progression of the disease. However, further studies are required to further confirm the beneficial effects described for extracts from *E. globulus* leaves in AD.

## Figures and Tables

**Table 1:** Chemical components present at ≥2%, of the essential oils from *Eucalyptus globulus* leaves.

Origin	Compounds	Source	Extraction	Yield (%)	References
Algeria	1,8-cineole (78.5 %), *o*-cymene (2.2%)	Fresh leaves	Steam distillation	0.96	[28]
	1,8-cineole (71.3 %), α-pinene (8.8%), *trans*-pinocarveol (3.3%), limonene (2.7%), α-terpineol (2.7%)	Fresh leaves	Steam distillation	1.10	[32]
	1,8-cineole (55.3%), isovaleraldehyde (10.0%), spathulenol (7.4%), α-terpineol (5.5%), α-pinene (4.6%)	Dry leaves	Hydrodistillation	2.53	[33]
	1,8-cineole (51.1%), α-pinene (24.6%), *trans*-pinocarveol (10.0%), globulol (2.8%)	Fresh leaves	Steam distillation	0.96	[34]
	1,8-cineole (48.6%), globulol (10.9%), *trans*-pinocarveol (10.7%), α-terpineol (6.6%), aromadendrene (4.6%)	Fresh leaves	Hydrodistillation	2.50	[35]
	1,8-cineole (47.1%), globulol (8.7%), α-pinene (7.7%), α-terpinene (3.6%), *p*-cymene (3.5%), terpineol (2.4%)	Dry leaves	Steam distillation	-	[36]
	γ-terpinene (94.5%), 1,8-cineole (3.2%)	Dry leaves	Hydrodistillation	3.50	[30]
Argentina	1,8-cineole (98.9%)	Commercial	-	-	[20]
	1,8-cineole (90.7%), α-pinene (4.1%)	Fresh leaves	Hydrodistillation	2.68	[37]
	^ a ^ 1,8-cineole (77.9%), α-terpineol (6.0%), α-pinene (5.8%), γ-terpinene (4.8%), *p*-cymene (2.3%)	Fresh leaves	Hydrodistillation	2.25	[38,39,40]
	^ b ^ 1,8-cineole (76.7%), α-pinene (11.1%), α-terpineol acetate (4.0%)			1.66	
	1,8-cineole (76.7%), limonene (18.9%)	Leaves	Hydrodistillation	-	[41]
Australia	1,8-cineole (90.0%), α-pinene (2.2%)	Commercial	-	-	[24]
	1,8-cineole (86.3%)	Commercial	-	-	[42]
	1,8-cineole (79.4%), α-pinene (3.7%), α-terpineol (3.0%)	Commercial	-	-	[43]
	1,8-cineole (77.0%), limonene (7.5%), *p*-cymene (5.5%), γ-terpinene (5.3%)	Commercial	-	-	[44]
	1,8-cineole (64.4%), limonene (5%), α-pinene (3.8%)	Commercial	-	-	[45]
	1,8-cineole (51.0%), α-pinene (16.7%), limonene (6.2%), globulol (7.3%)	Leaves	Hydrodistillation	-	[46]
Belgium	1,8-cineole (80.4%), limonene, (7.5%), γ-terpinene (3.7%), *p*-cymene (2.5%)	Commercial	-	-	[47]
Brazil	1,8-cineole (90.0%)	Commercial	-	-	[48]
	1,8-cineole (90.0%), tricyclene (3.0%)	Commercial	-	-	[23]
	1,8-cineole (83.9%), limonene (8.2%), α-pinene (4.2%), *o*-cymene (3.0%)	Commercial	-	-	[49]
	1,8-cineole (83.7%), limonene (6.4%), *p*-cymene (5.4%), α-pinene (4.6%)	Fresh leaves	Hydrodistillation	-	[50]
	1,8-cineole (78.9%), limonene (8.5%), *p*-cymene (5.7%), α-pinene (3.6%)	Commercial	-	-	[51]
	1,8-cineole (77.5%), α-pinene (14.2%)	Dry leaves	Hydrodistillation	3.10	[52]
	1,8-cineole (75.7%), *p*-cymene (7.5%), α-pinene (7.3%), limonene (6.4%)	Commercial	-	-	[53,54]
	1,8-cineole (71.0%), α-pinene (8.3%), α-guaiene (4.8%), globulol (3.5%), *cis*-verbenol (2.7%)	Dry leaves	Steam distillation	1.33	[55]
	1,8-cineole (69.3%), camphene (9.4%), α-pinene (7.5%), α-terpineol (5.1%), globulol (2.7%)	Dry leaves	Hydrodistillation	1.60	[56]
	1,8-cineole (68.3%), α-pinene (16.2%), α-terpineol (6.4%), limonene (3.0%)	Dry leaves	Steam distillation	-	[57]
	1,8-cineole (64.3%), α-pinene (8.9%), α-terpineol (7.2%), globulol (4.8%)	Dry leaves	Hydrodistillation	1.60	[58]
	1,8-cineole (61.3%), camphenene (12.6%), α-pinene (5.8%), limonene (4.1%), vidiflorol (3.1%), aromadrendene (2.8%)	Dry leaves	Hydrodistillation	1.50	[59]
	1,8-cineole (49.0%), camphenene (8.9%), globulol (7.0%), aromadendrene (2.3%), α-terpineol (2.0%)	Dry leaves	Hydrodistillation	0.60	[60]
	1,8-cineole (44.7%), α-pinene (14.3%), globulol (9.2%), aromadendrene (7.3%), *p*-cymene (4.7%)	Dry leaves	Hydrodistillation	-	[61]
Cameroon	1.8-cineole (26.4%), α-pinene (14.1%), *p*-cymene (10.2%), β-ionone epoxyde (7.0%), *p*-menthen-8-ol (6.5%)	Fresh leaves	Steam distillation	1.00	[62]
Chile	1,8-cineole (82.6%), α-pinene (9.5%), m-mentha-6.8-diene (4.7%)	Leaves	Hydrodistillation	-	[63]
	1,8-cineole (76.0%), α-pinene (7.4%), aromadendrene (3.6%), silvestrene (2.8%), sabinene (2.0%)	Fresh leaves	Hydrodistillation	-	[64]
China	1,8-cineole (94.3%)	Commercial	-	-	[65]
	1,8-cineole (39.2%), α-terpineol acetate (13.8%), α-terpineol (11.3%), α-pinene (11.3%), *endo*-borneol (5.4%)	Dry leaves	Hydrodistillation	-	[66]
Columbia	1,8-cineole (52.3%), α-pinene (15.3%), α-terpineol (9.8%), globulol (7.6%)	Fresh leaves	Hydrodistillation	1.50	[67]
Democratic Republic of the Congo	1,8-cineole (44.3%), camphene (23.1%), α-pinene (9.3%), globulol (7.3%), limonene (5.1%)	Fresh leaves	Hydrodistillation	1.87	[68]
Ecuador	1,8-cineole (52.6%), α-pinene (20.0%), α-phellandrene (6.2%), α-terpinyl acetate (3.7%)	Commercial	-	-	[69]
Egypt	1,8-cineole (46.8%), limonene (9.6%), tolueno (8.6%), *o*-cymene (6.5%), fenchene (6.3%)	Dry leaves	Hydrodistillation	-	[70]
	1,8-cineole (21.4%), *o*-cymene (21.4%), α-pinene (6.7%), spathulenol (6.3%), 4-terpineol (3.9%)	Fresh leaves	Hydrodistillation	0.40	[71]
Ethiopia	1,8-cineole (81.6%), α-pinene (2.8%), cuminaldehyde (2.8%), *trans*-caryophyllene (2.5%)	Fresh leaves	Hydrodistillation	-	[26]
	1,8-cineole (63.0%), α-pinene (16.1%), camphor (3.4%)	Fresh leaves	Hydrodistillation	-	[72]
	1,8-cineole (57.5%), α-pinene (15.2%), limonene (7.8%), α-terpinyl acetate (5.3%), α-terpineol (2.0%)	Fresh leaves	Hydrodistillation	1.10	[73]
France	1,8-cineole (57.9%), α-pinene (13.9%), globulol (3.6%), *p*-cymene (3.3%), *trans*-pinocarveol (2.8%)	Commercial	-	-	[74]
Germany	1,8-cineole (86.5%), α-pinene (4.7%), γ-terpinene (2.6%)	Commercial	-	-	[75]
India	1,8-cineole (85.0%), α-pinene (3.0%)	Commercial		-	[25]
	1,8-cineole (71.7%), α-pinene (9.14%), α-terpineol acetate (3.6%), alloaromadendrene (2.4%), α-terpineol (2.2%)	Dry leaves	Hydrodistillation	-	[76]
	1,8-cineole (71.6%), 3-carene (15.1%), *cis*-ocimene (6.2%)	Commercial	-	-	[77]
	1,8-cineole (68.8%), α-pinene (2.8%), *p*-cymene (2.1%)	Commercial	-	-	[78]
	1,8-cineole (54.8%), β-pinene (18.5%), α-pinene (11.5%), β-eudesmol (4.7%), α-phellandrene (2.1%),	Fresh leaves	Hydrodistillation	1.10	[79]
	1,8-cineole (66.3%), *cis*-ocimene (21.3%), α-terpinyl acetate (3.4%), aromadendrene (2.9%), globulol (1.4%)	Commercial	-	-	[80]
	1,8-cineole (33.6%), α-pinene (14.2%), limonene (10.1%), α-terpinolene (6%), α-terpineol (4.7%)	Commercial	-	-	[81]
	1,8-cineole (45.4%), limonene (17.8%), *p*-cymene (9.5%), γ-terpinene (8.8%), α-pinene (4.2%)	Commercial	-	-	[82]
	*p*-cymene (31.9%), 1,8-cineole (17.5%), α-pinene (17.2%), α-terpinene (8.9%), β-pinene (7.5%)	Fresh leaves	Hydrodistillation	0.90	[83]
	cymene (26.4%), β-pinene (15.2%), eudesmol (11.4%), α-pinene (10.6%), 1-phellandrene (10.3%)	Dry leaves	Hydrodistillation	2.00	[31]
Iran	1,8-cineole (88.0%), α-pinene (2.2%)	Dry leaves	Hydrodistillation	-	[84]
	1,8-cineole (58.1%), α-phellandrene (6.0%), neo-isodihydrocarveol (3.6%), α-pinene (3.3%), α-eudesmol (3.2%)	Commercial	-	-	[85]
Italy	1,8-cineole (95.5%), α-pinene (2.5%)	Commercial	-	-	[22]
	1,8-cineole (91.5%), *p*-cymene (3.1%), α-pinene (2.7%)	Commercial	-	-	[86]
	1,8-cineole (89.8%), *p*-cymene (6.7%), α-pinene (2.0%)	Commercial	-	-	[87,88,89]
	1,8-cineole (84.9%), α-pinene (5.6%), *p*-cymene (5.3%)	Commercial	-	-	[90]
	1,8-cineole (81.4%), limonene (7.0%)	Commercial	-	-	[91,92]
	1,8-cineole (76.0%), α-pinene (6.6%), limonene (5.7%), α-terpineol (3.1%)	Commercial	-	-	[93]
	1,8-cineole (48.2%), aromadendrene (13.7%), guaiol (7.6%), α-pinene (6.9%), *p*-mentha-1,3,5-triene (3.8%)	Dry leaves	Hydrodistillation	2.00	[94]
Kenya	1,8-cineole (79.6%), α-pinene (6.9%), α-terpineol (3.8%), limonene (2.7%)	Fresh leaves	Hydrodistillation	-	[26]
	1,8-cineole (17.2%), α-pinene (7.1%), spathulenol (6.5%), cryptone (5.4%), isoborneol (2.5%)	Fresh leaves	Steam distillation	-	[95]
Montenegro	1,8-cineole (85.8%), α-pinene (7.2%)	Dry leaves	Hydrodistillation	1.80	[96]
Morocco	1,8-cineole (80.0%), limonene (6.7%), *p*-cymene (5.1%), γ-terpinene (3.9%)	Dry leaves	Steam distillation	2.70	[27]
	1,8-cineole (70.6%), α-pinene (12.9%)	Dry leaves	Hydrodistillation	0.60	[97]
	1,8-cineole (29.5%), *p*-cymene (11.5%), α-terpineol (5.2%)	Dry leaves	Hydrodistillation	1.20	[98]
	*p*-cymene (37.8%), 1,8-cineole (29.3%), limonene (26.1%), α-pinene (3.5%)	Commercial	-	-	[99]
Pakistan	1,8-cineole (56.5%), limonene (28.0%), α-pinene (4.2%) α-terpineol, (4.0%), globulol (2.4%)	Fresh leaves	Hydrodistillation	1.89	[100]
	β-phellandrene (32.1%), 1,8-cineole (26.6%), α-pinene (16.8%), *p*-cymene (8.9%), Δ^3^-carene (8.1%)	Fresh leaves	Hydrodistillation	1.10	[101]
Portugal	1,8-cineole (74.6%), α-pinene (12.9%), metileugenol (3.5%), globulol (3.2%), terpinen-4-ol (2.0%)	Dry leaves	Hydrodistillation	-	[29]
	1,8-cineole (62.5%), α-pinene (18.5%), limonene (4.0%) aromadendrene (3.1%), δ-cadinene (2.9%)	Fresh leaves	Hydrodistillation	1.90–2.70	[102]
	1,8-cineole (36.7%), β-pinene (9.3%), aromedendrene (6.3%), globulol (5.1%), *trans*-pinocarveol (2.5%)	Dry leaves	Hydrodistillation	2.67	[103]
Slovakia	1,8-cineole (70.0%), limonene (12.0%), α-pinene (9.0%)	Leaves	Hydrodistillation	-	[104]
South Africa	1,8-cineole (80.8%), limonene (8.0%), γ-terpinene (2.8%)	Commercial	-	-	[105]
Spain	1,8-cineole (84.3%), cymene (7.5%), γ-terpinene (3.5%)	Commercial	-	-	[106]
	1,8-cineole (63.8%), α-pinene (16.1%), aromadendrene (3.7%), *o*-cymene (2.4%)	Commercial	-	-	[107]
Switzerland	1,8-cineole (88.0%), *p*-cymene (6.7%), γ-terpinene (3.5%)	Commercial	-	-	[108]
Thailand	1,8-cineole (82.6%), limonene (7.4%), *o*-cymene (4.3%), γ-terpinene (2.7%)	Commercial	-	-	[109]
	1,8-cineole (48.5%), α-pinene (20.6%), β-pinene (15.5%), terpineol (15.4%)	Fresh leaves	Hydrodistillation	-	[110]
Tunisia	1,8-cineole (95.6%)	Commercial	-	-	[21]
	1,8-cineole (62.8%), 4-methyl-2-pentyl acetate (22.3%), α-pinene (8.8%), caryophyllene (2.5%), β-humulene (2.4%)	Commercial	-	-	[111]
	1,8-cineole (53.8%), α-pinene (12.1%), globulol (4.5%), *trans*-pinocarveol (3.7%), aromadendrene (3.4%)	Dry leaves	Hydrodistillation	3.80	[112]
	1,8-cineole (48.2%), α-pinene (16.1%), γ-terpinene (8.9%), *p*-cymene (8.8%), globulol (3.8%)	Fresh leaves	Hydrodistillation	0.74	[113]
	1,8-cineole (43.2%), α-pinene (13.6%), aromadendrene (10.1%), 4-carene (6.9%), β-cymene (4.0%)	Dry leaves	Hydrodistillation	1.25	[114]
	*p*-cymene (18.2%), methyl eugenol (8.8%), terpinenol (8.5%), s-methyl 3-methylbutanethioate (7.3%), γ-terpinene (5.1%)	Fresh leaves	Steam distillation	-	[115]
USA	1,8-cineole (90.0%), *p*-cymene (3.7%), α-pinene (3.5%)	Commercial	-	-	[116]

^a^*E. globulus* ssp. *maidenii*; ^b^* E. globulus* ssp. *globulus*.

**Table 2:** Phenolic compounds from *Eucalyptus globulus* leaves extracts.

Origin	Compounds * mg/100 g Plant Material	Total Phenolic Content mg GAE/g	Source	Extraction	Yield g/100 g Plant Material	Reference
Algeria	Sideroxylonal (1902.39), ellagic acid (284.30), methylellagic acid hexose (174.88), eucalbanine (113.13), quercetin 3-O-rhamnoside (108.43)	-	Dry leaves	70% Acetone and 0.5% acetic acid	24.70	[118]
Australia	Hyperoside (66.64), quercetin (28.78), myricetin (9.23), rutin (4.87), isoquercetin (3.90)	235.87 ^a^	Dry leaves	70% Ethanol at 60 °C	-	[121]
Chile	Luteolin (260.00), quercetin (250.00), morin (170.00), sinapic acid (170.00), ellagic acid (60.00)	0.043 ^b^	Fresh leaves	Methanol	-	[64]
	Gallic acid (2175.00), gentisic acid (1358.33), rutin (456.83), caffeic acid (351.67), 3,4-dihydroxybenzoic acid (34.33)	54.02 ^c^	Dry leaves	Water at 100 °C		[122]
China	Rutin, isorhamnetin-hexoside, isorhamnetin-rhamnoside	-	Dry leaves	Methanol at 45 °C	10.50	[123]
Egypt	Isorhamnetin 3-*O*-beta-D-glucuronoside, galloyl cypellocarpin B, cypellocarpin C, methyl gallate, valoneoyl-digalloyl-glucopyranose	-	Dry leaves	Methanol	14.67	[124]
Greece	*p*-Coumaric acid (6.60), quercetin (2.50), rutin (1.80), gallic acid (1.50)	-	Dry leaves	62.5% Methanol and HCl at 90 °C	-	[125]
India	Gallic acid (8.62), ellagic acid (6.58), vanillic acid (4.89), *p*-hydroxybenzoic acid (4.36), syringic acid (3.86)	242.50 ^c^	Dry leaves	Methanol	-	[126]
	Gallic acid (5.36), ellagic acid (4.20), *p*-hydroxybenzoic acid (3.55), vanillic acid (2.56), syringic acid (2.45)	156.30 ^c^		Chloroform		
	Gallic acid (3.08), *p*-hydroxybenzoic acid (2.10), syringic acid (1.24), ellagic acid (0.86)	98.70 ^c^		Hexane		
	Rutin (113.20), quercetin (44.00), ferulic acid (6.66), gallic acid (3.00), caffeic acid (1.40)	40.10 ^c^	Dry leaves	80% Methanol and 5.5% HCl at 85 °C	-	[127]
Lithuania	Chlorogenic acid, phlorizin, rutin, quinic acid, isoquercetin	-	Dry leaves	70% Methanol	-	[13]
	Chlorogenic acid, phlorizin, rutin, quinic acid, isoquercetin	-		70% Acetone		
	Chlorogenic acid, phlorizin, quinic acid, quercetin, apigenin	-		70% Ethanol		
Portugal	Flavonol glycoside (234.52), chlorogenic acid (106.91), rutin (105.71), ellagic acid (63.81), quercetin (57.38)	311.00 ^c^	Dry leaves	Water at 40 °C	23.80	[128]
Spain	Hyperoside (29.09), chlorogenic acid (17.54), rutin (16.64), quercetin (6.30), *p*-coumaric acid derivative (2.40)	-	Dry leaves	Water	9.69	[129]
USA	Gallic acid (132.90)	-	Dry leaves	50% Methanol	30.00	[130]

* Only the five major compounds from highest to lowest concentration are shown in the table; ^a^ value expressed in dry weight plant material; ^b^ value expressed in fresh weight plant material; ^c^ value expressed in extract.

## Data Availability

Not applicable.
